# Supplementary material for: Serum Bile Acids Concentrations and Liver Enzyme Activities after Low-Dose Trilostane in Dogs with Hyperadrenocorticism
Source: Animals (Basel). 2023 Oct 18;13(20):3244. doi: 10.3390/ani13203244 (PMC10603711; doi:10.3390/ani13203244)
Supplement: Supplementary file 1 [file animals-13-03244-s001.zip › animals-2648192-SI.pdf]

Table S1. Liver enzymes in dogs with HAC before and after supplementation with hepatoprotectants.

Parameter (Reference Range)	Duration of supplemention [Median (range); days]	Before supplementation with hepatoprotectants (N=15)	After supplemention with hepatoprotectants (N=15)	*p*-value
ALT (6–70 U/L)	60 (22 to 367)	188 (27 to 1045)	239 (27 to 937)	0.2078
ALP (8–76 U/L)		963 (218 to 7798)	2049 (303 to 8438)*	0.0067

ALT, alanine aminotransferase; ALP, alkaline phosphatase
